# Monitoring of postoperative neutrophil-to-lymphocyte ratio, D-dimer, and CA153 in: Diagnostic value for recurrent and metastatic breast cancer

**DOI:** 10.3389/fsurg.2022.927491

**Published:** 2023-01-06

**Authors:** Zhiyao Ren, Jing Yang, Jiahui Liang, Yunfeng Xu, Guanda Lu, Yanxun Han, Jie Zhu, Husheng Tan, Tao Xu, Min Ren

**Affiliations:** ^1^ Department of Breast Surgery, The First Affiliated Hospital of Anhui Medical University, Hefei, China; ^2^ Department of General Surgery, The First Affiliated Hospital of Anhui Medical University, Hefei, China; ^3^ First Clinical Medical College, Anhui Medical University, Hefei, China; ^4^ Department of Otolaryngology, Head and Neck Surgery, The First Affiliated Hospital of Anhui Medical University, Hefei, Anhui Province, China; ^5^ Department of Infectious Diseases, The First Affiliated Hospital of Anhui Medical University, Hefei, China; ^6^ School of Pharmacy, Anhui Medical University, Hefei, China

**Keywords:** recurrence and metastasis, breast cancer, neutrophil-to-lymphocyte ratio, d-dimer, carbohydrate antigen 153

## Abstract

**Objective:**

This stydy aims to assess the value of monitoring of postoperative neutrophil-to-lymphocyte ratio (NLR), D-dimer, and carbohydrate antigen 153 (CA153) for diagnosis of breast cancer (BC) recurrence and metastasis.

**Materials/Methods:**

A cohort of 252 BC patients who underwent surgery at the First Affiliated Hospital of Anhui Medical University between August 2008 and August 2018 were enrolled in this retrospective study. All patients were examined during outpatient follow-ups every 3 months for 5 years postoperation and every 6 months thereafter. Recurrence or metastasis was recorded for 131 patients but not for the remaining 121. Retrospective analysis of hematological parameters and clinicopathological characteristics allowed comparison between the two groups and evaluation of these parameters for the recurrent and metastatic patients.

**Results:**

Lymph node metastasis, higher tumor node metastasis (TNM) staging, and higher histological grade correlated with BC recurrence and metastasis (*p* < 0.05). Statistical differences were found in absolute neutrophil count (ANC), absolute lymphocyte count (ALC), CEA, CA153, D-dimer, NLR, platelet-to-lymphocyte ratio (PLR), and monocyte-to-lymphocyte ratio (MLR) between the recurrent and metastatic and control groups (*p* < 0.05). Logistic regression analysis showed that CA153, D-dimer, NLR, and TNM staging were risk factors for BC recurrence and metastasis (*p* < 0.05). Combined values for the NLR, D-dimer, and CA153 had good diagnostic values, giving the highest area under the curve (AUC) of 0.913. High NLR, D-dimer, and CA153 values were significantly associated with recurrence and metastasis at multiple sites, lymph node metastasis, and higher TNM staging (*p* < 0.05). Patients with high CA153 were more likely to have bone metastases (*p* < 0.05), and those with high D-dimer were prone to lung metastasis (*p* < 0.05). With the increasing length of the postoperative period, the possibility of liver metastases gradually decreased, while that of chest wall recurrence gradually increased (*p* < 0.05).

**Conclusion:**

Monitoring postoperative NLR, D-dimer, and CA153 is a convenient, practical method for diagnosing BC recurrence and metastasis. These metrics have good predictive value in terms of sites of recurrence and metastasis and the likelihood of multiple metastases.

## Introduction

Breast cancer (BC) has the highest incidence rate worldwide of any cancer type (1). Prognosis has shown improvement in response to large-scale promotion of physical examination and advances in treatment (2). However, 20%–30% of BC patients suffer from recurrence and metastasis, contributing significantly to mortality (3–5). Therefore, timely postoperative monitoring for recurrence and metastasis to allow initiation of appropriate treatment is of great value. Emerging screening methods, such as circulating tumor cell (CTC) measurements, assist with diagnosing recurrence and metastasis (6) but are limited by expense and equipment requirements. A biopsy is the main approach, but its practicality for screening is limited due to its invasiveness. Therefore, there is an urgent need for hematological indices that are cheap, easy, and noninvasive to assist clinicians in the diagnosis of BC recurrence and metastasis in the postoperative period.

There is an increasing awareness of the role played by inflammation in the development and progress of many diseases, including cancer (7, 8). Indeed, hematological parameters used to evaluate systemic inflammation could have prognostic value for various cancers (9, 10). Commonly used hematological parameters include absolute neutrophil count (ANC), absolute lymphocyte count (ALC), absolute monocyte count (AMC), absolute platelet (PLT) count, red blood cell distribution width (RDW), platelet distribution width (PDW), and ratios generated from these data, such as neutrophil-to-lymphocyte ratio (NLR), platelet-to-lymphocyte ratio (PLR), and monocyte-to-lymphocyte ratio (MLR). Furthermore, tumor markers have great diagnostic and prognostic value (11, 12), with CEA and CA153 commonly used to diagnose BC recurrence and metastasis (13, 14). Moreover, activation of the coagulation system and fibrinolytic cascade may correlate with tumor growth, invasion, and metastasis, and plasma D-dimer is a procoagulation factor that may reflect micrometastases (15–17). A high D-dimer level is considered to reflect a poor prognosis for a variety of cancers (17–19).

The current study comprehensively compared the above 12 hematological parameters, namely, ANC, ALC, AMC, RDW, PLT, PDW, NLR, PLR, MLR, CEA, CA153, and D-dimer, with clinical features. The aim was to determine the most useful combination of hematological parameters for accurate diagnosis of recurrence and metastasis in postoperative BC patients. It is hoped that biomarker identification will assist in detection and early treatment, improving the survival rate and quality of life of recurrent and metastatic BC patients.

## Materials and methods

### Study participants

A cohort of 252 BC patients who underwent surgery at the First Affiliated Hospital of Anhui Medical University between August 2008 and August 2018 was retrospectively enrolled. The final follow-up date was December 1, 2021: patients classified as having recurrence and metastasis were diagnosed before this date, while patients in the control group had no recurrence or distant metastasis by this date. All patients were examined during outpatient follow-ups every 3 months for the first postoperative 5 years and every 6 months thereafter. Recurrence or metastasis was diagnosed in 131 patients within 10 years by biopsy, PET-CT, or radionuclide bone imaging during re-examination (recurrent and metastatic group). The remaining 121 patients showed no recurrence or metastasis (control group). No differences in age at diagnosis, history of smoking, history of drinking, body mass index (BMI), place of residence, menopause status, interval, and systematic treatment history, including operation, chemotherapy, or hormone therapy, were found between the two groups (*p* > 0.05; Table 1). The “interval” is defined as the time between the operation and the first diagnosis of recurrence or metastasis (recurrent and metastatic group) or the time between the operation and the final re-examination of the study (control group).

**Table 1:** Demographic characteristics and systematic treatment history of the two groups.

Demographic characteristics and systematic treatment history	Recurrent and metastatic group (*n* = 131)	Control group (*n* = 121)	Total	*χ*^2^/*t*	*p*
Age (mean ± SD), years	51.02 ± 10.46	50.80 ± 9.44	50.91 ± 9.96	0.175	0.861
History of smoking, *n* (%)					
Yes	14 (10.69%)	7 (5.79%)	21 (8.33%)	1.949	0.160
No	117 (89.31%)	114 (94.21%)	231 (91.67%)		
History of drinking, *n* (%)					
Yes	85 (64.89%)	73 (60.33%)	158 (62.70%)	0.558	0.455
No	46 (35.11%)	48 (39.67%)	94 (37.30%)		
Body mass index (kg/m^2^)	21.44 ± 3.18	20.85 ± 4.22	21.45 ± 5.07	1.259	0.209
Place of residence, *n* (%)					
Countryside	82 (62.60%)	70 (57.85%)	152 (60.32%)	0.591	0.442
City	49 (37.40%)	51 (42.15%)	100 (39.68%)		
Menopause, *n* (%)					
Yes	68 (51.91%)	70 (57.85%)	138 (54.76%)	0.879	0.344
No	63 (48.09%)	51 (42.15%)	114 (45.24%)		
Operation, *n* (%)					
Breast conserving surgery	46 (35.11%)	41 (33.88%)	87 (34.52%)	0.042	0.837
Modified radical mastectomy	85 (64.89%)	80 (66.12%)	165 (65.48%)		
Chemotherapy, *n* (%)					
None	16 (12.21%)	10 (8.26%)	26 (10.32%)	3.768	0.583
AC-Taxans	42 (32.06%)	44 (36.36%)	86 (34.13%)		
AC	28 (21.37%)	26 (21.49%)	54 (21.43%)		
TAC	10 (7.63%)	8 (6.61%)	18 (7.14%)		
FAC	29 (22.14%)	22 (18.18%)	51 (20.24%)		
Other	6 (4.58%)	11 (9.09%)	17 (6.75%)		
Hormone therapy, *n* (%)					
None	40 (30.53%)	38 (31.40%)	78 (30.95%)	2.999	0.223
Tamoxifen	62 (47.33%)	46 (38.02%)	108 (42.86%)		
Aromatase inhibitor	29 (22.14%)	37 (30.58%)	66 (26.19%)		
Interval, *n* (%)					
<1 year	19 (14.50%)	10 (8.26%)	29 (11.51%)	2.564	0.277
1–4 years	78 (59.54%)	80 (66.12%)	158 (62.70%)		
5–10 years	34 (25.95%)	31 (25.62%)	65 (25.79%)		

AC, adriamycin and cyclophosphamide; FAC, fluorouracil, adriamycin, and cyclophosphamide; TAC, taxan, adriamycin, and cyclophosphamide.

Inclusion criteria are as follows: a postoperative pathological diagnosis of BC; complete clinical data; and no recurrence or metastasis shown by B-mode ultrasound and CT before the operation. Exclusion criteria are as follows: nonsurgical treatment; incomplete clinical data; co-occurrence of severe heart, liver, kidney, or infectious diseases or other malignant tumors; abnormal PET-CT imaging brought about by abscesses or active infection; and recent administration of drugs affecting bone metabolism.

This study was approved by the Ethics Committee of the First Affiliated Hospital of Anhui Medical University (reference number: PJ 2021-10-32).

### Methods

Postoperative pathological results, hematological parameters, and other data, such as demographic characteristics and clinical data, were compared between the two groups.

All postoperative specimens were analyzed by the Department of Pathology at our hospital. Hormone receptor status, Ki-67, and human epidermal growth factor receptor 2 (HER2) status were assessed by immunohistochemical analysis. Positive immunohistochemical identification of estrogen and/or progesterone receptors led to a hormone receptor-positive classification. HER2 positivity was defined as an immunohistochemistry result of 3+ or 2+, subsequently confirmed by in situ fluorescent hybridization experiments. Molecular subtypes of BC were categorized as luminal A-like, luminal B-like, HER-2, or triple-negative breast cancer (TNBC) on the basis of Ki-67 and HER2 status, according to St Gallen 2013 recommendations.

Blood samples were collected at each follow-up re-examination. Hematological indexes were derived from the re-examination in which patients were first diagnosed with recurrence and metastasis (recurrent and metastatic group) or the final postoperative re-examination (control group). Patients’ fasting venous blood samples were collected in the morning and placed in vacuum blood collection tubes. Serum CA153 and CEA were determined by a multitumor marker protein chip diagnostic kit (Huzhou Shukang Biotechnology), and D-dimer was determined by a latex immunoturbidimetry kit (Beijing Lidman) with detection by a Hitachi 7080 automatic biochemical analyzer. Routine blood tests, including ANC, ALC, AMC, PLT count, RDW, and PDW, were performed by an automated hematology analyzer (XN9000, Sysmex, Japan) according to the manufacturer’s instructions. NLR was calculated by dividing ANC by ALC, PLR was calculated by dividing PLT by ALC, and MLR was calculated by dividing AMC by ALC.

### Statistical analysis

Statistical analysis was performed using SPSS 25.0 software. Measurement data were expressed as mean ± standard deviation (SD). Student’s *t*-test and Mann–Whitney *U* test were used to compare two groups of data, with *t*-test results checked by the Shapiro–Wilk test for normally distributed data or the Mann–Whitney *U* test results checked for non-normally distributed data. One-way analysis of variance (ANOVA) was used to compare more than two groups. Count data were expressed as [*n* (%)] and compared using the *χ*^2^ test. Receiver operating characteristic (ROC) curve analysis was performed to calculate the area under the curve (AUC) to determine diagnostic efficacy. Sensitivity, specificity, and cutoff value for each index were determined according to the maximum Youden index. Multivariate logistic regression was used to analyze independent risk factors for the diagnosis of BC recurrence and metastasis. Results were considered statistically significant when *p* < 0.05.

## Results

### Clinicopathological characteristics

Postoperative pathological information from the two groups of patients was subjected to univariate analysis. There were no statistically significant differences in pathological types or molecular subtypes between the two groups (*p* > 0.05) but significant differences in lymph node metastasis, TNM staging, and histological grade (*p* < 0.05; Table 2). The locations and numbers of sites of recurrence and metastasis are shown in Table 2. Bone (43.51%), lung (32.06%), and liver (23.66%) were the most frequent sites.

**Table 2:** Clinicopathological characteristics of the two groups.

Clinicopathological characteristics	Recurrent and metastatic group (*n* = 131)	Control group (*n* = 121)	Total	*χ* ^2^	*p*
Pathological type, *n* (%)					
Invasive carcinoma	115 (87.78%)	102 (84.30%)	217 (86.11%)	4.349	0.114
Intraductal carcinoma	2 (1.53%)	8 (6.61%)	10 (3.97%)		
Other	14 (10.69%)	11 (9.09%)	25 (9.92%)		
Lymph node metastasis, *n* (%)					
Yes	92 (70.23%)	48 (39.67%)	140 (55.56%)	23.791	0.000
No	39 (29.77%)	73 (60.33%)	112 (44.44%)		
TNM staging, *n* (%)					
I + II	25 (19.08%)	66 (54.55%)	91 (36.11%)	34.285	0.000
III	106 (80.92%)	55 (45.45%)	161 (63.89%)		
Molecular subtype, *n* (%)					
HER-2	13 (9.92%)	14 (11.57%)	27 (10.71%)	2.017	0.569
Luminal A	36 (27.48%)	33 (27.27%)	69 (27.38%)		
Luminal B	41 (31.30%)	45 (37.19%)	86 (34.13%)		
TNBC	41 (31.30%)	29 (23.97%)	70 (27.78%)		
Histologic grade, *n* (%)					
I	6 (4.58%)	19 (15.7%)	25 (9.92%)	22.983	0.000
II	53 (40.46%)	69 (57.02%)	122 (48.41%)		
III	72 (54.96%)	33 (27.27%)	105 (41.67%)		
Sites of recurrence and metastasis, *n* (%)					
Bone	57 (43.51%)	–	–		
Lung	42 (32.06%)	–	–		
Liver	31 (23.66%)	–	–		
Brain	23 (17.56%)	–	–		
Chest wall (recurrence)	17 (12.98%)	–	–		
Other	18 (13.74%)	–	–		
Number of sites, *n* (%)					
Single	91 (69.47%)	–	–		
Multiple	40 (30.53%)	–	–		

Other metastatic sites refer to pleural metastasis, peritoneal metastasis, and adrenal metastasis.

Number of sites indicates the number of sites with recurrence and/or metastasis. Thereinto, patients with recurrence and metastasis also belong to “multiple.” TNM, tumor node metastasis; TNBC, triple-negative breast cancer.

### Comparison and ROC analysis of hematological indicators between the two groups

**Figure 1:**
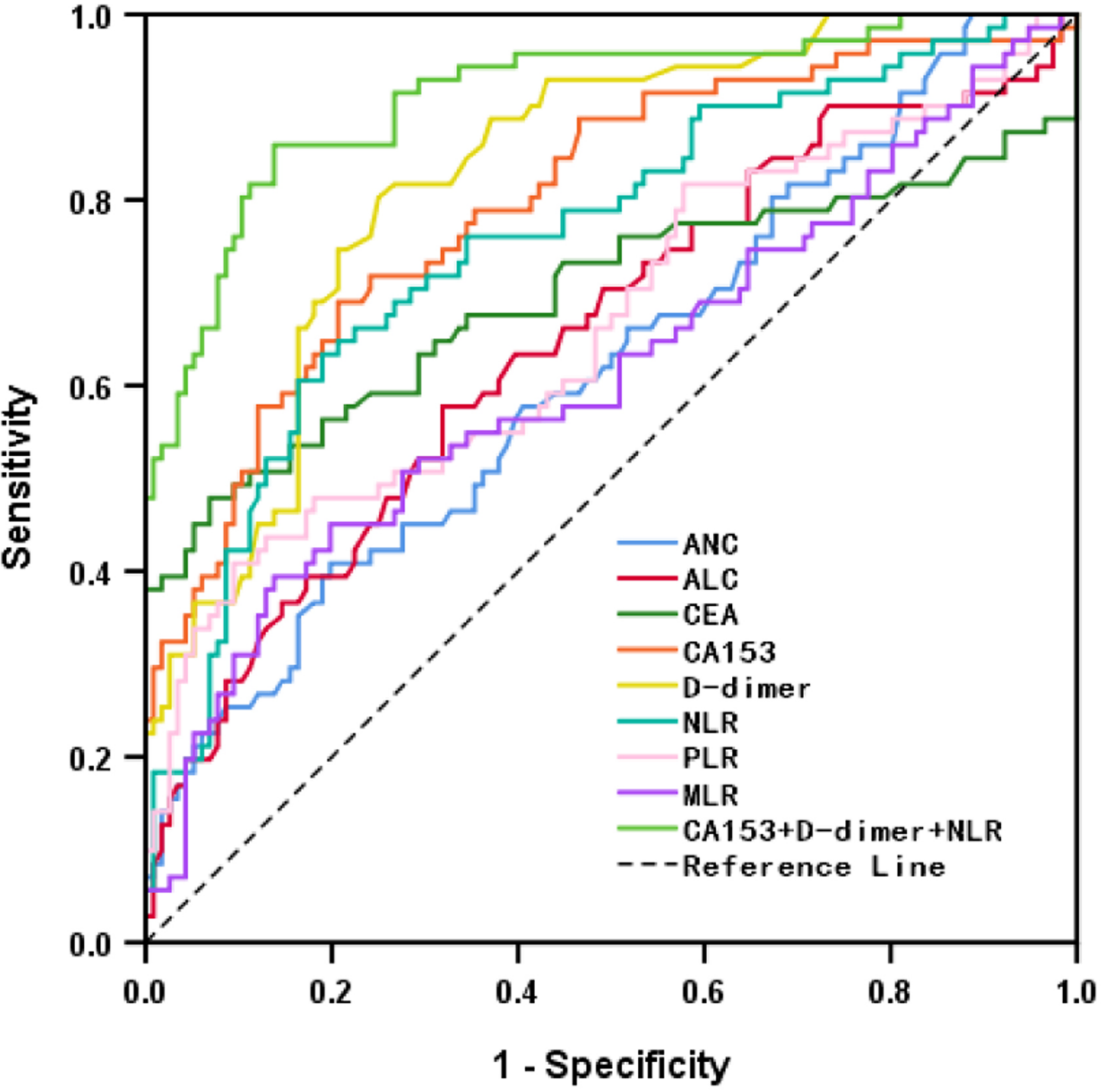
ROC of hematological indicators for diagnosing recurrence and metastasis of breast cancer.

The hematological indicators, ANC, ALC, CEA, CA153, D-dimer, NLR, PLR, and MLR, showed statistically significant differences (*p* < 0.05) but AMC, RDW, PLT, and PDW did not (*p* > 0.05; Table 3). ROC curves were plotted and AUCs were calculated for selected markers to assess the diagnostic value for BC recurrence and metastasis. D-dimer was the most promising, giving the highest AUC value of 0.828 (CI, 0.769–0.887) and sensitivity of 0.803. CEA gave the highest specificity of 0.931, followed by PLR with 0.905 (Table 4 and Figure 1).

**Table 3:** Comparison of hematological parameters in the two groups.

Variables	Recurrent and metastatic group (*n* = 131)	Control group (*n* = 121)	*Z*	*p*
ANC (×10^9^/L)	4.18 ± 1.78	3.36 ± 1.22	−3.490	0.000
ALC (×10^9^/L)	1.46 ± 0.66	1.69 ± 0.68	−2.994	0.003
AMC (×10^9^/L)	0.36 ± 0.15	0.34 ± 0.14	−0.750	0.453
RDW (fl)	43.90 ± 4.06	43.68 ± 5.18	−0.278	0.781
PLT (×10^9^/L)	211.03 ± 60.04	205.51 ± 64.80	−1.233	0.217
PDW (fl)	14.03 ± 4.14	13.20 ± 2.86	−1.929	0.054
CEA (ng/ml)	32.66 ± 142.21	2.01 ± 0.97	−4.451	0.000
CA153 (KU/L)	45.89 ± 64.90	13.73 ± 9.39	−5.409	0.000
D-dimer (µg/ml)	2.00 ± 3.29	0.49 ± 0.49	−7.198	0.000
NLR	3.63 ± 2.91	2.23 ± 1.19	−4.824	0.000
PLR	171.25 ± 85.55	132.81 ± 50.74	−3.402	0.001
MLR	0.30 ± 0.21	0.23 ± 0.12	−2.787	0.005

ANC, absolute neutrophil count; ALC, absolute lymphocyte count; CEA, carcinoembryonic antigen; PLR, platelet-to-lymphocyte ratio; MLR, monocyte-to-lymphocyte ratio; AMC, absolute monocyte count; PDW, platelet distribution width; RDW, red blood cell distribution width; PLT, platelet; NLR, neutrophil-to-lymphocyte ratio.

**Table 4:** Diagnostic values of hematological indicators for recurrence and metastasis of breast cancer.

Variables	AUC (95%CI)	Sensitivity	Specificity	Youden’s index	Cut-off
ANC	0.619 (0.535–0.702)	0.408	0.801	0.21	4.08
ALC	0.650 (0.568–0.733)	0.577	0.681	0.258	0.727
CEA	0.695 (0.606–0.784)	0.479	0.931	0.41	3.66
CA153	0.799 (0.733–0.866)	0.69	0.793	0.483	19.48
D-dimer	0.828 (0.769–0.887)	0.803	0.75	0.553	0.49
NLR	0.758 (0.686–0.830)	0.634	0.81	0.444	2.841
PLR	0.664 (0.581–0.748)	0.408	0.905	0.314	193.475
MLR	0.614 (0.528–0.701)	0.394	0.862	0.256	0.3
CA153 + D-dimer + NLR	0.913 (0.869–0.957)	0.861	0.862	0.723	3.159

ANC, absolute neutrophil count; ALC, absolute lymphocyte count; CEA, carcinoembryonic antigen; PLR, platelet-to-lymphocyte ratio; MLR, monocyte-to-lymphocyte ratio; NLR, neutrophil-to-lymphocyte ratio.

### Multivariate analysis and diagnostic value of combined detection for BC recurrence and metastasis

**Figure 2:**
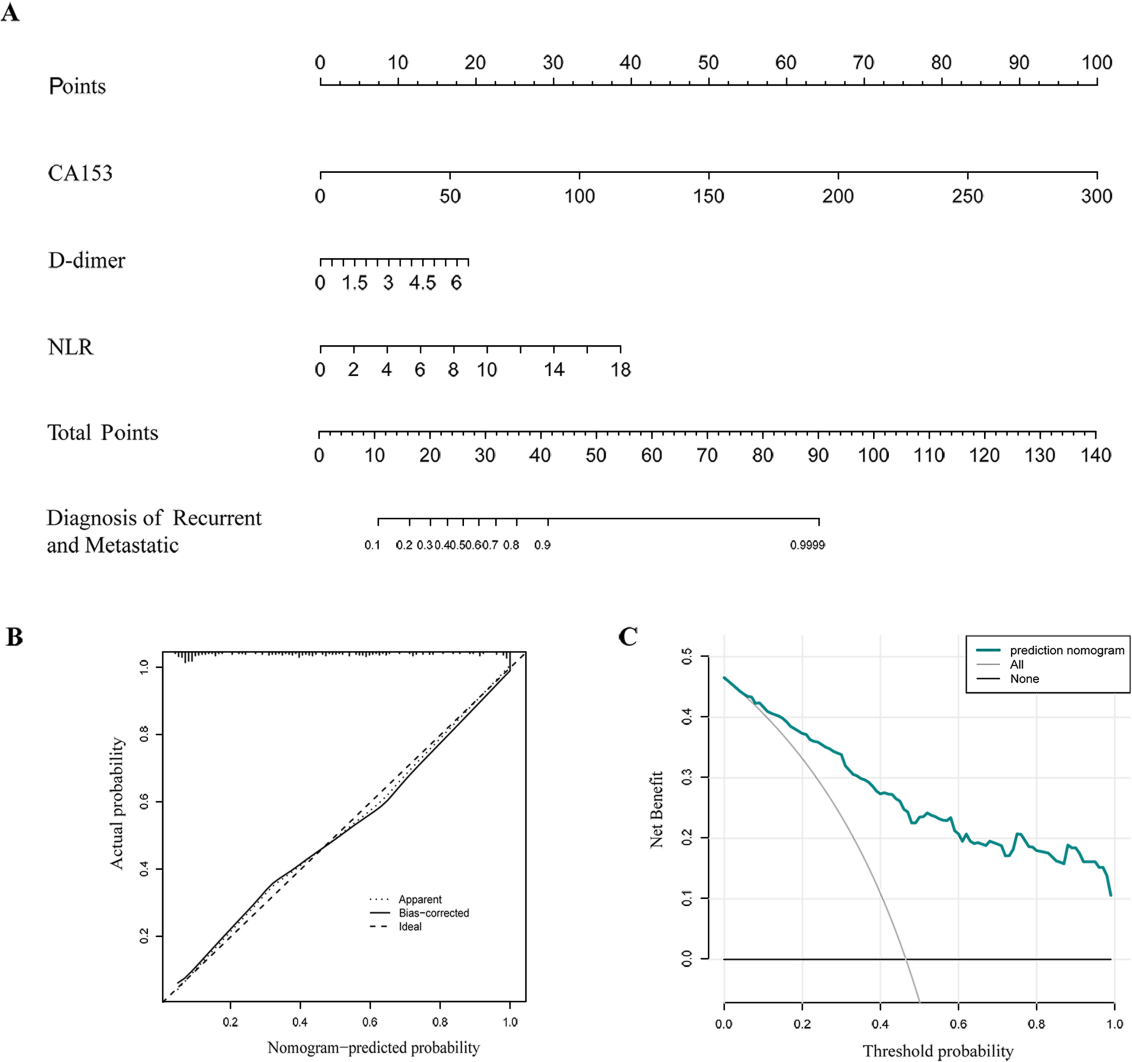
Nomogram for calculating the risk of recurrence and metastasis in breast cancer patients (**A**). Calibration curve (**B**). Decision curve analysis (DCA) (**C**).

Lymph node metastasis, TNM staging, histological grade, ANC, ALC, CEA, CA153, D-dimer, NLR, PLR, and MLR were selected from the above univariate analysis and assigned values (Table 5). Multivariate logistical regression analysis showed that CA153, D-dimer, NLR, and TNM staging were risk factors for BC recurrence and metastasis (Table 6; *p* < 0.05). Values were assigned to CA153, D-dimer, and NLR (not shown in the table) for multivariate logistical regression analysis (Supplementary Table S1) to derive a formula for combined detection with these three markers. The formula was *Y *= −4.049 + (0.066CA153) + (1.181D-dimer) + (0.499NLR). The diagnostic value of combined detection was also assessed (Table 4 and Figure 1). The AUC was 0.913 (0.869–0.957), sensitivity was 0.861, specificity was 0.862, and the cut-off value was 3.159. Moreover, we constructed a nomogram integrating the three markers to calculate the total score of each patient (Figure 2A), which is the sum of the scores of the above risk factors. Total score = Point (CA153) + Point (D-Dimer) + Point (NlR), and predict the possibility of breast cancer recurrence and metastasis according to the total score. The calibration plot (Figure 2B) showed that the predicted and observed proportions of breast cancer recurrence and metastasis were very similar. We further performed decision curve analysis (DCA) (Figure 2C), showing that the nomogram showed a good net benefit for clinical utility and could be used as an auxiliary tool for diagnosing BC recurrence and metastasis. Combined detection exhibited better diagnostic value than any single hematological indicator and has potential utility as an adjuvant tool for diagnosing BC recurrence and metastasis. It is noteworthy that, although CEA and PLR showed the highest specificities, they were not risk factors for BC recurrence and metastasis by multivariate analysis, suggesting inferior diagnostic values to those of CA153, D-dimer, and NLR. No statistical differences in levels of CA153, D-dimer, and NLR were found between BC patients with recurrence and those with metastasis (Supplementary Table S2; *p* > 0.05), and no heterogeneity in these three indices was found. Thus, the two groups of patients were combined for the following analysis.

**Table 5:** Assignment.

Factor	Value
Lymph node metastasis	No = 0; Yes = 1
TNM staging	I + II = 1; III = 2
Histologic grade	I = 1; II = 2; III = 3
ANC	Continuous data variables using raw data analysis
ALC	Continuous data variables using raw data analysis
CEA	Continuous data variables using raw data analysis
CA153	Continuous data variables using raw data analysis
D-dimer	Continuous data variables using raw data analysis
NLR	Continuous data variables using raw data analysis
PLR	Continuous data variables using raw data analysis
MLR	Continuous data variables using raw data analysis

ANC, absolute neutrophil count; ALC, absolute lymphocyte count; CEA, carcinoembryonic antigen; PLR, platelet-to-lymphocyte ratio; MLR, monocyte-to-lymphocyte ratio; NLR, neutrophil-to-lymphocyte ratio.

**Table 6:** Multivariate analysis of risk factors for recurrence and metastasis in breast cancer patients.

Variables	B	SE	Wald	*p*	OR	OR 95% CI
CA153	0.024	0.009	7.111	0.008	1.024	1.009–1.443
D-dimer	0.835	0.302	7.645	0.006	2.305	1.861–3.572
NLR	0.584	0.227	6.169	0.010	1.793	1.131–2.950
TNM staging	0.317	0.144	4.846	0.028	1.373	1.184–2.140
Constant	−0.623	0.212	8.636	0.003	0.536	

B, constant term; SE, standard error; Wald, *χ*^2^ value; OR, odds ratio.

### Association of NLR, Ca153, D-dimer, and clinicopathological characteristics for the recurrent and metastatic group

Optimal cutoff values (Table 4) were used to subdivide the recurrent and metastatic group into those with high and low NLR, CA153, and D-dimer levels for analysis of relationships with clinicopathological characteristics (Table 7). High NLR, CA153, and D-dimer were positively associated with multiple recurrent and metastatic sites, lymph node metastasis, and higher TNM staging (*p* < 0.05) but were not related to the remaining clinical characteristics. Moreover, the number of sites was not affected by interval (Supplementary Table S3).

**Table 7:** Relationship between NLR, CA153, D-dimer, and clinicopathological characteristics of the recurrent and metastatic group.

Clinicopathological characteristics		NLR		CA153 (U/ml)		D-dimer (µg/ml)	
		<2.841	≥2.841	<19.480	≥19.480	<0.490	≥0.490
Age (years)	<60	42 (61.76%)	36 (57.14%)	38 (61.29%)	40 (57.97%)	46 (64.79%)	32 (53.33%)
	≥60	26 (38.24%)	27 (42.86%)	24 (38.71%)	29 (42.03%)	25 (35.21%)	28 (46.67%)
*χ*^2^		0.290		0.149		1.771	
*p*		0.590		0.699		0.183	
Interval (years)	<1	12 (17.65%)	7 (11.11%)	10 (16.13%)	9 (13.04%)	13 (18.31%)	6 (10.00%)
	1–4	41 (60.29%)	37 (58.73%)	37 (59.68%)	41 (59.42%)	40 (56.34%)	38 (63.33%)
	5–10	15 (22.06%)	19 (30.16%)	15 (24.19%)	19 (27.54%)	18 (25.35%)	16 (26.67%)
*χ*^2^		1.803		0.355		1.837	
*p*		0.406		0.837		0.399	
Number of sites, *n* (%)	single	53 (77.94%)	38 (60.32%)	49 (79.03%)	42 (60.87%)	57 (80.28%)	34 (56.67%)
	multiple	15 (22.06%)	25 (39.68%)	13 (20.97%)	27 (39.13%)	14 (19.72%)	26 (43.33%)
χ^2^		4.789		5.079		8.550	
* p*		0.029		0.024		0.003	
Lymph node metastasis, *n* (%)	Yes	40 (58.82%)	52 (82.54%)	34 (54.84%)	58 (84.06%)	44 (61.97%)	48 (80.00%)
	No	28 (41.18%)	11 (17.46%)	28 (45.16%)	11 (15.94%)	27 (38.03%)	12 (20.00%)
χ^2^		8.797		13.335		5.055	
*p*		0.003		0.000		0.025	
TNM staging, *n* (%)	I + II	18 (26.47%)	7 (11.11%)	19 (30.65%)	6 (8.70%)	22 (30.99%)	3 (5.00%)
	III	50 (73.53%)	56 (88.89%)	43 (69.35%)	63 (91.30%)	49 (69.01%)	57 (95.00%)
χ^2^		4.996		10.189		14.220	
* p*		0.025		0.001		0.000	
Molecular subtype, n (%)	HER2+	8 (11.76%)	5 (7.94%)	7 (11.29%)	6 (8.70%)	9 (12.68%)	4 (6.67%)
	Luminal A	21 (30.88%)	15 (23.81%)	19 (30.65%)	17 (24.64%)	20 (28.17%)	16 (26.67%)
	Luminal B	19 (27.94%)	22 (34.92%)	22 (35.48%)	19 (27.54%)	24 (33.80%)	17 (28.33%)
	TNBC	20 (29.41%)	21 (33.33%)	14 (22.58%)	27 (39.13%)	18 (25.35%)	23 (38.33%)
χ^2^		1.748		4.167		3.272	
*p*		0.626		0.244		0.352	
Histologic grade, *n* (%)	I	4 (5.88%)	2 (3.17%)	3 (4.84%)	3 (4.35%)	5 (7.04%)	1 (1.67%)
	II	29 (42.65%)	24 (38.10%)	27 (43.55%)	26 (37.68%)	31 (43.66%)	22 (36.67%)
	III	35 (51.47%)	37 (58.73%)	32 (51.61%)	40 (57.97%)	35 (49.30%)	37 (61.67%)
χ^2^		1.005		0.535		3.350	
*p*		0.605		0.765		0.187	

### Association of different sites of recurrence and metastasis with NLR, Ca153, D-dimer, and clinicopathological characteristics

Comparisons of different sites of recurrence and metastasis with NLR, CA153, D-dimer, and clinicopathological characteristics in the recurrent and metastatic group are presented in Table 8. More bone metastases were found in the high CA153 group than in the low CA153 group (*χ*^2 ^= 6.065, *p* = 0.014). The rate of lung metastasis was higher in the high D-dimer group than that in the low D-dimer group (*χ*^2 ^= 4.689, *p* = 0.03). The interval was also related to the positive rates of bone and lung metastases (bone: *χ*^2 ^= 12.986, *p* = 0.002; lung: *χ*^2 ^= 7.589, *p* = 0.022). With increased intervals, the rate of liver metastases gradually decreased, while that of chest wall recurrence gradually increased.

**Table 8:** Association of different sites of recurrence and metastasis with NLR, CA153, DD, and clinicopathological characteristics.

Variables		Bone metastases		Lung metastases		Liver metastases		Brain metastases		Recurrence (chest wall)	
		No	Yes	No	Yes	No	Yes	No	Yes	No	Yes
NLR	<2.841	41 (60.3%)	27 (39.7%)	50 (73.5%)	18 (26.5%)	54 (79.4%)	14 (20.6%)	58 (85.3%)	10 (14.7%)	58 (85.3%)	10 (14.7%)
	≥2.841	33 (52.4%)	30 (47.6%)	39 (61.9%)	24 (38.1%)	46 (73.0%)	17 (27.0%)	50 (79.4%)	13 (20.6%)	56 (88.9%)	7 (11.1%)
χ^2^		0.83		2.03		0.74		0.79		0.37	
*p*		0.361		0.154		0.389		0.373		0.541	
CA153 (U/ml)	<19.480	42 (67.7%)	20 (32.3%)	44 (71.0%)	18 (29.0%)	45 (72.6%)	17 (27.4%)	53 (85.5%)	9 (14.5%)	55 (88.7%)	7 (11.3%)
	≥19.480	32 (46.4%)	37 (53.6%)	45 (65.2%)	24 (34.8%)	55 (79.7%)	14 (20.3%)	55 (79.7%)	14 (20.3%)	59 (85.5%)	10 (14.5%)
χ^2^		6.065		0.496		0.919		0.752		0.297	
*p*		0.014		0.481		0.338		0.386		0.586	
DD (µg/ml)	<0.490	44 (62.0%)	27 (38.0%)	54 (76.1%)	17 (23.9%)	55 (77.5%)	16 (22.5%)	59 (83.1%)	12 (16.9%)	62 (87.3%)	9 (12.7%)
	≥0.490	30 (50.0%)	30 (50.0%)	35 (58.3%)	25 (41.7%)	45 (75.0%)	15 (25.0%)	49 (81.7%)	11 (18.3%)	52 (86.7%)	8 (13.3%)
χ^2^		1.896		4.689		0.109		0.046		0.012	
*p*		0.168		0.03		0.741		0.83		0.911	
Interval (years)	<1	12 (63.2%)	7 (36.8%)	10 (52.6%)	9 (47.4%)	9 (47.4%)	10 (52.6%)	16 (84.2%)	3 (15.8%)	18 (94.7%)	1 (5.3%)
	1–4	44 (56.4%)	34 (43.6%)	58 (74.4%)	20 (25.6%)	60 (76.9%)	18 (23.1%)	65 (83.3%)	13 (16.7%)	71 (91.0%)	7 (9.0%)
	5–10	18 (52.9%)	16 (47.1%)	21 (61.8%)	13 (38.2%)	31 (91.2%)	3 (8.8%)	27 (79.4%)	7 (20.6%)	25 (73.5%)	9 (26.5%)
χ^2^		0.518		4.115		12.986		0.300		7.589	
*p*		0.772		0.128		0.002		0.861		0.022	
TNM staging	I + II	16 (64.0%)	9 (36.0%)	18 (72.0%)	7 (28.0%)	20 (80.0%)	5 (20.0%)	22 (88.0%)	3 (12.0%)	22 (88.0%)	3 (12.0%)
	III	58 (54.7%)	48 (45.3%)	71 (67.0%)	35 (33.0%)	80 (75.5%)	26 (24.5%)	86 (81.1%)	20 (18.9%)	92 (86.8%)	14 (13.2%)
χ^2^		0.709		0.234		0.23		0.659		0.026	
*p*		0.4		0.629		0.632		0.417		0.872	
Histologic grade	I	4 (66.7%)	2 (33.3%)	3 (50.0%)	3 (50.0%)	5 (83.3%)	1 (16.7%)	4 (66.7%)	2 (33.3%)	4 (66.7%)	2 (33.3%)
	II	30 (56.6%)	23 (43.4%)	40 (75.5%)	13 (24.5%)	45 (84.9%)	8 (15.1%)	48 (90.6%)	5 (9.4%)	46 (86.8%)	7 (13.2%)
	III	40 (55.6%)	32 (44.4%)	46 (63.9%)	26 (36.1%)	50 (69.4%)	22 (30.6%)	56 (77.8%)	16 (22.2%)	64 (88.9%)	8 (11.1%)
χ^2^		0.279		2.809		4.21		4.53		2.426	
*p*		0.87		0.245		0.122		0.104		0.297	
Lymph node metastasis	Yes	49 (53.3%)	43 (46.7%)	60 (65.2%)	32 (34.8%)	67 (72.8%)	25 (27.2%)	74 (80.4%)	18 (19.6%)	78 (84.8%)	14 (15.2%)
	No	25 (64.1%)	14 (35.9%)	29 (74.4%)	10 (25.6%)	33 (84.6%)	6 (15.4%)	34 (87.2%)	5 (12.8%)	36 (92.3%)	3 (7.7%)
χ^2^		0.848		1.051		2.107		0.861		1.373	
*p*		0.357		0.305		0.147		0.354		0.241	

NLR, neutrophil-to-lymphocyte ratio; TNBC, triple-negative breast cancer.

## Discussion

Several previous reports have established hematological indicators as risk factors with prognostic value for BC. However, most studies have only focused on pretreatment levels of one indicator or a class of indicators, and further work was required to identify the most effective diagnostic indicators. In addition, pretreatment hematological indicators undoubtedly have prognostic value for cancer patients, but hematological indices are influenced by many factors with a physiological impact on patients, including treatment, lifestyle, and environmental factors. Improvement of the diagnostic potential requires refined attention to the level of hematological indicators at the point of diagnosis of recurrence and metastasis, which better represents the physiological state of patients at this point. Recurrence and metastasis can occur at any point, and monitoring is needed to account for changes in hematological indicators with changes in the patient’s physiological state. We believe that this is the first study to combine commonly used prognostic indicators producing a comprehensive analysis for selecting the best-performing diagnostic indicators, paying attention to the diagnostic rather than prognostic value for BC recurrence and metastasis.

Statistical differences in lymph node metastasis, TNM staging, histological grade, ANC, ALC, CEA, CA153, D-dimer, NLR, PLR, and MLR were found between patients in the recurrent and metastatic group and those in the control group. Multivariate analysis identified CA153, D-dimer, NLR, and TNM staging as independent risk factors for diagnosis of recurrence and metastasis. Few studies have been conducted on D-dimer and the prognosis of BC. However, the current study found that D-dimer has the highest AUC and sensitivity by comparison with tumor markers and indices of systemic inflammation. Previous studies have established activated coagulation and thrombosis as common in malignancy with relevance to metastasis (20, 21). Indeed, Dirix et al. (17) found that D-dimer levels were increased in nearly 89% of patients with metastatic BC and levels correlated with CTCs (22). Currently, insufficient clinical attention is given to the coagulation index in outpatient re-examination of BC patients. The current study indicated the high clinical value of D-dimer for diagnosing BC recurrence and metastasis, making this index worthy of promotion. Further exploration of other coagulation indicators is also merited. NLR (ANC/ALC) is considered an indicator of systemic inﬂammation (23). Neutrophils are reported to promote the invasion and metastasis of tumor cells, remodeling of the extracellular matrix, and angiogenesis (24) and have been associated with detrimental outcomes of several solid tumors (25). By contrast, lymphocytes act to kill cancer cells and improve clinical outcomes in various cancers (25–27). It may be concluded that the ratio of these two indicators better reflects the tumor-related inflammatory response. Whereas NLR has been associated with the prognosis of various cancers (28–31), its value as a diagnostic index for recurrent and metastatic breast cancer has not been previously investigated. Moldoveanu et al. (32) found higher NLRs among triple-negative breast cancer patients during the 6 months prior to diagnosis of recurrence, suggesting increased NLR before or after BC recurrence and metastasis. These observations indicate the value of monitoring NLR after treatment to aid in early diagnosis of recurrence and metastasis. The current work has shown higher NLRs after recurrence and metastasis than controls. CA153 has been shown previously to be a good diagnostic indicator for BC metastasis and superior to CEA (13, 14, 33–35). Such findings are consistent with our conclusions. Moreover, the current research also demonstrated the good diagnostic value of CA153 in BC recurrence. It is worth noting that although CEA and PLR had low sensitivity and were excluded from the multivariate analysis, their high specificity can be used to judge suspected recurrent and metastatic BC. Combined detection of CA153, D-dimer, and NLR was confirmed as an excellent diagnostic index for recurrent and metastatic BC (AUC = 0.913). The calibration curve and the decision curve demonstraed that the nomogram integrating the three indexes can accurately diagnose recurrence and metastasis of BC patients.

BC often metastasizes to bone, lung, liver, and soft tissue (36). Different levels of indicators may give locational information. The current study established a higher positive rate of CA153 in bone metastases than for other sites. Many previous studies have indicated the value of CA153 in diagnosing bone metastases in BC patients, consistent with our findings (35, 37, 38). However, Yerushalmi et al. (39) did not find significant differences in CA153 between different sites of metastasis. The current work identified the positive rate of D-dimer as more associated with lung metastases than other metastatic sites. No previous literature has reported a relationship between D-dimer and lung metastases. We may infer that hypercoagulability and thrombosis are important factors inducing lung metastasis of breast cancer. The study of Park et al. (40) demonstrated that 4T1 BC cells injected into the mouse tail vein could be found within mouse lungs and caused the formation of a neutrophil extracellular trap, a significant factor causing thrombosis in cancer patients (21). This experiment establishes a potential relationship between thrombosis and lung metastasis. Based on the above results, we reiterate our view that D-dimer should be an indicator included in BC outpatient re-examination. In addition, with increasing postoperative time, the frequency of liver metastases gradually decreased, while that of chest wall recurrence gradually increased. Late recurrence should thus be anticipated by clinicians. However, the small sample size of the current study means that this result should be interpreted cautiously. The study conducted by Moon et al. (41) found that NLR analysis performed 5 years after the primary diagnosis predicted late BC recurrence. A comparison of patients suffering recurrence and metastasis after 5 years with those suffering within 5 years found no significant differences in NLR, D-dimer, and CA153. Therefore, we believe that NLR, D-dimer, and CA153 should be monitored even in those patients without recurrence and metastasis within 5 years. High NLR, CA153, and D-dimer were also found to be significantly associated with multiple recurrent and metastatic sites, lymph node metastasis, and higher TNM staging. Previous studies have reported higher CA153 and D-dimer in BC patients with multiple metastatic sites than in those with single metastasis (17, 42–44). Significantly elevated CA153, NLR, and D-dimer levels observed in BC patients with multiple recurrent and metastatic sites may reflect tumor burden.

We acknowledge some limitations to the current study. First, BC is a highly heterogeneous disease and a single-center retrospective study with a relatively small sample size may have limited accuracy. However, the inclusion of varied pathological types and molecular subtypes and the assurance of homogeneity between the two groups should minimize the interference caused by heterogeneity. We will continue to collect samples in the future to further improve this research. Second, the hematological parameters could be influenced by pathophysiological conditions, and we matched patients’ basic information and treatment history to minimize the effects of other factors. Third, this study focused on the value of NLR, D-dimer, and CA153 in diagnosing recurrent and metastatic BC. However, it lacks an external validation cohort; prospective multicenter studies and a validation cohort will be designed to refine the findings.

## Conclusion

The current single-center retrospective study conﬁrmed the value of CA153, D-dimer, NLR, and TNM staging as independent predictors of recurrent and metastatic BC. Combined detection of CA153, D-dimer, and NLR yielded the greatest diagnostic value. The same indicators also had utility in predicting numbers and locations of recurrent and metastatic sites. To the best of our knowledge, this study is the first to provide comprehensive insights into a series of hematological parameters for diagnosing recurrent and metastatic BC.

## Data Availability

The raw data supporting the conclusions of this article will be made available by the authors without undue reservation.
