# Supplementary material for: Monitoring of postoperative neutrophil-to-lymphocyte ratio, D-dimer, and CA153 in: Diagnostic value for recurrent and metastatic breast cancer
Source: Front Surg. 2023 Jan 6;9:927491. doi: 10.3389/fsurg.2022.927491 (PMC9853451; doi:10.3389/fsurg.2022.927491)
Supplement: Supplementary file 1 [file Datasheet1.docx]

**TABLE S1 |** Multivariate analysis of CA153, D-dimer and NLR for recurrence and metastasis in breast cancer patients.

| **Variables** | **B** | **SE** | **Wald** | **P** | **OR** | **OR 95% CI** |
| --- | --- | --- | --- | --- | --- | --- |
| CA153 | 0.066 | 0.019 | 11.861 | 0.001 | 1.069 | 1.029-1.981 |
| D-dimer | 1.181 | 0.429 | 7.595 | 0.006 | 3.258 | 1.407-5.339 |
| NLR | 0.499 | 0.160 | 9.729 | 0.002 | 1.648 | 1.204-3.185 |
| Constant | -4.049 | 0.653 | 38.431 | 0.000 | 0.017 |  |

B: constant term, SE: standard error, Wald: chi-square value, OR: odds ratio.

**TABLE S2** **|** Comparison of CA153, D-dimer and NLR between the recurrent group and metastatic group.

| **Variables** | **CA153** | **D-dimer** | **NLR** |
| --- | --- | --- | --- |
| Recurrent Group | 49.93±59.68 | 1.38±1.24 | 2.82±1.53 |
| Metastatic Group | 45.20±66.02 | 2.10±3.52 | 3.75±3.05 |
| Z | -0.818 | -0.203 | -1.157 |
| P | 0.413 | 0.839 | 0.247 |

**TABLE S3 |** Comparison of number of sites between different intervals of recurrent and metastatic group.

| **Interval(years)** | **N** | **Number of sites (n)** | **F** | **P** |
| --- | --- | --- | --- | --- |
| ＜1 | 19 | 1.78±1.39 | 1.402 | 0.249 |
| 1-4 | 78 | 1.42±0.71 |  |  |
| 5-10 | 34 | 1.53±0.75 |  |  |
